# Interpretable modeling for material transportation process in an industrial cement vertical roller mill

**DOI:** 10.1038/s41598-026-66824-3

**Published:** 2026-08-14

**Authors:** Rasoul Fatahi, Hadi Abdollahi, Mohammad Noaparast, Saeed Chehreh Chelgani

**Affiliations:** 1 School of Mining Engineering, College of Engineering, University of Tehran, Tehran, Iran; 2 Minerals and Metallurgical Engineering, Department of Civil, Environmental and Natural Resources Engineering, Swedish School of Mines, Luleå University of Technology, Luleå, Sweden

**Keywords:** Modeling, Conscious lab, Explainable artificial intelligence, Material transportation, CatBoost, Energy science and technology, Engineering, Mathematics and computing

## Abstract

Vertical roller mills (VRMs) dominate cement finish grinding because of their high efficiency and dry operation. However, the interaction among ventilation, fan operation, and pneumatic material transport remains poorly quantified using plant data. In this study, the previously established data-driven Conscious Lab (*CL*) framework, based on explainable machine learning, is developed to model material transport behavior in an industrial VRM circuit at the Tehran Cement Plant. A total of 1050 operating records collected over eight months under diverse conditions were cleaned and analyzed. Three ensemble algorithms (CatBoost, XGBoost, and Random Forest) were trained to predict two transport-relevant targets, mill fan speed and mill fan power, and were assessed with conventional hold-out splits and nested cross-validation to reduce selection bias. Among the evaluated models, CatBoost delivered the most reliable performance, achieving high predictive accuracy for fan speed (${R}^{2}\sim 0.97$) and fan power (${R}^{2}\sim 0.95$). Interpretability was ensured through SHAP analysis, which ranked the dominant drivers of each target and revealed nonlinear interactions among differential pressure, grinding pressures, water injection, and feed-related responses. The results highlight the important role of ventilation-related variables, particularly mill fan speed, fan power, and differential pressure, in governing pneumatic transport and circuit stability. The proposed *CL* framework provides an explainable, operator-oriented tool to support process diagnosis, setpoint decisions, and future development of real-time intelligent control strategies for energy-efficient cement grinding.

## Introduction

Vertical roller mills (VRMs) are high-efficiency grinding circuits commonly used in the cement and coal processing industries. These mills are considered more energy-efficient in cement than traditional tumbling mills^1^. Over 80% of new cement mills in the cement industry are VRMs, offering greater energy efficiency and flexibility than conventional ball mills^2^. VRMs are preferred for final cement grinding over other mills due to their higher capacity, simplified process, lower energy consumption, and compact design^3^. These mills integrate four key functions: material transportation, grinding, drying, and classification into a single instrument^4^. The grinding process in the VRMs is controlled and adjusted by altering several operational variables, such as working pressure, feed rate, table speed, and differential pressure (dp), among others^5,6^. The mill’s performance is influenced by its design, which dictates a specific operational range for feed rates and throughputs^7^.

In current practice, operators primarily assess a VRM's operational status by monitoring parameter changes and adjusting based on experience. In other words, traditional VRM control systems in cement plants rely primarily on field staff manually adjusting process parameters based on practice. Relying heavily on operator experience can make maintaining stable operation under changing process conditions challenging, potentially leading to fluctuations in process stability, energy consumption, and production efficiency, as reported in previous studies^8^. Although the foundational principles of regulation are well-established, the empirically proposed strategies are often difficult to implement effectively^7^. These facts are essential since the control and optimization of VRM circuits have become increasingly important due to their widespread use in industrial applications, and evaluating the performance of these mills is crucial to effectively manage the system and enhance its efficiency^9^. Some studies have examined VRMs to highlight the importance of understanding and modeling interactions among VRM operational variables and their performance. They generally considered two main points: modeling comminution with breakage behavior within the mill and optimizing process control.

On the mechanistic side, researchers have measured and fitted residence time distributions (RTDs) in industrial clinker VRMs using classic RTD structures (e.g., dispersion, tanks-in-series, and mixer–bypass concepts) to better describe internal transport and mixing, which are essential for predicting grinding response^10^. They have also simulated size reduction using population-balance and matrix-model frameworks, estimating breakage functions from laboratory tests and demonstrating multi-stage pressure breakage behavior within VRMs^11^. Extending this, a “number of breakages” viewpoint was used to link mean residence time to repeated breakage events, improving understanding of how VRM operating conditions translate into clinker breakage intensity^12^.

**Fig. 1:**
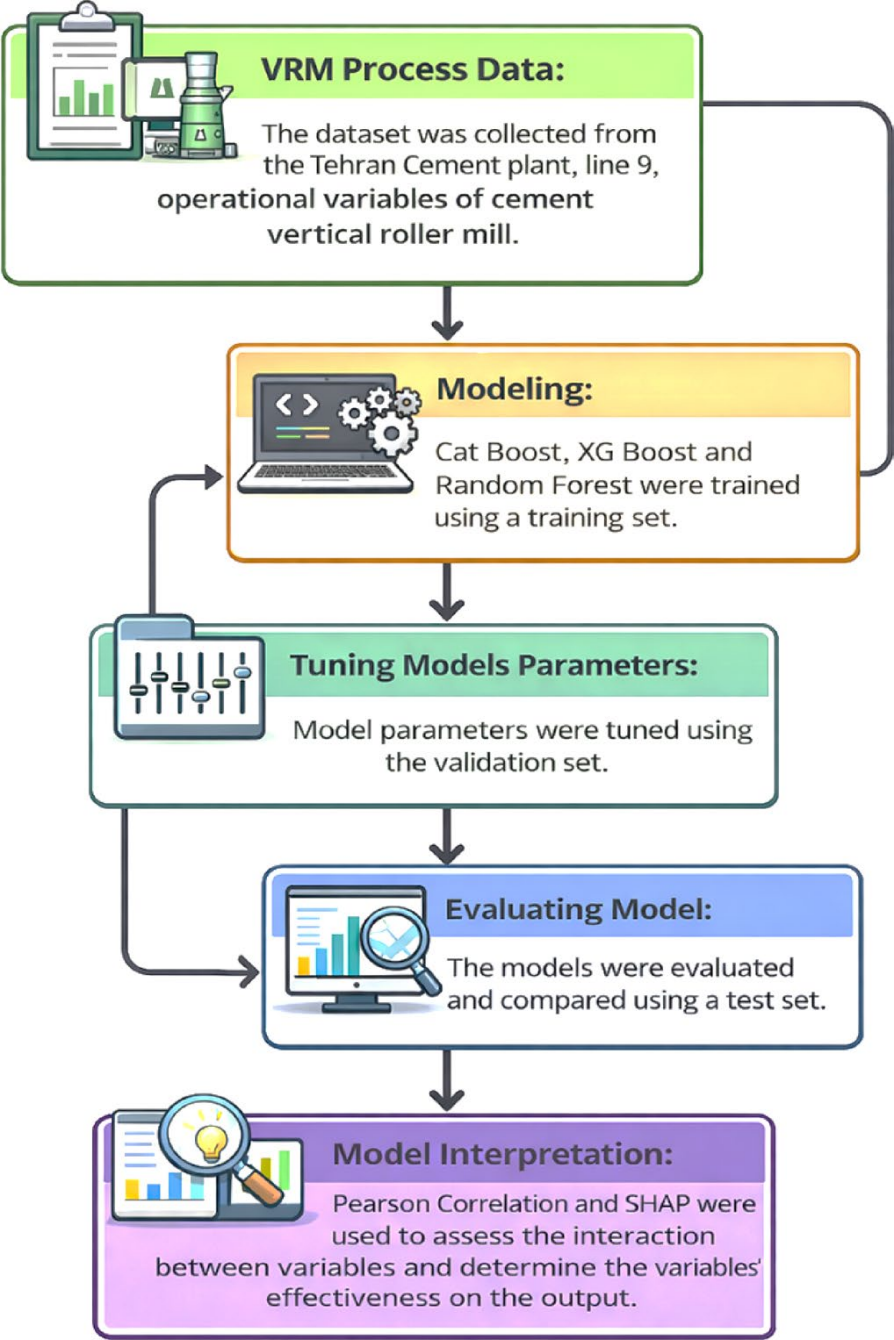
The methodology used to construct the CL for a VRM with the SHAP-CatBoost model.

However, few investigations considered new advanced modeling systems. Fatahi et al.^13^, by considering the conscious lab “*CL*” concept (using advanced AI modeling for industrial data assessment (Fig. 1)), developed a data-driven and control-oriented work that targeted soft sensing, prediction, and optimization: examples include an explainable ML (SHAP-XGBoost) model to rank and interpret the operational drivers of VRM. Shaoming and Chao^14^ generated an intelligent control system for slag (GGBS) vertical milling built around data-driven condition evaluation to support optimal control actions, and neural-network soft sensors for grinding circuits, such as a GRNN fineness estimator built from plant data^15^. Finally, the same “conscious lab” and explainable AI philosophy has been applied to adjacent cement-unit operations (e.g., ventilation prediction in raw ball mills and rotary kiln variable modeling), illustrating how interpretable AI can map complex interactions among variables to support operational decision-making^16^.

As an essential VRM operational parameter, the optimal operation of the mill fan is a key component of the ventilation system, helping maintain VRM stability and productivity. Proper fan adjustment ensures uniform material flow, prevents accumulation and blockages, and enhances material transportation throughout the circuit. Conversely, any ventilation malfunction or improper fan settings directly lead to increased vibrations, frequent mill trips, and a noticeable reduction in production capacity, thereby significantly increasing energy consumption. Industrial operational evidence demonstrates a strong interdependence between fan operational parameters (such as speed and power) and both material transportation efficiency and the overall quality of the grinding process^17–19^. Therefore, optimal control of these variables is essential for achieving a stable circuit with efficient energy use. Despite the mill fan's critical importance, most previous research has focused primarily on improving grinding parameters. While VRMs operate in a dry system^20–22^, previous *CL*-based investigations have focused primarily on grinding-related process variables, including working pressure, feed rate, differential pressure, and main drive power. However, the ventilation-driven material-transportation subsystem, governed primarily by mill fan operation, has not previously been investigated using this explainable modeling framework. Addressing these knowledge gaps and establishing a sustainable, long-term production process are critical.

Therefore, this study will explore the relationships between operational parameters and their impacts on mill fan operation, leading to the development of a *CL* model that enables optimal control of the VRM grinding circuit, improves circuit efficiency, and facilitates its operation with deeper insight and a more informed approach. The main aim of this study is to develop a *CL* for the VRM at the Tehran Cement Plant in Tehran Province, Iran, using the SHAP-CatBoost model as an interpretable tool. The study will also evaluate the VRM's performance in terms of its material transportation response (mill fan power and speed), which is influenced by various operational parameters. Unlike previous CL investigations^16–19^ that focused on grinding-related responses (working pressure, feed rate, differential pressure, and main drive power), the present study addresses the ventilation-driven material transportation subsystem of the VRM.

## Materials and methods

### Data collection

A dataset was collected from the central control room of Line 9 at Tehran Cement Plant, based on daily monitoring of the VRM (Variable Speed Motor) circuit over an 8-month period (from March 10, 2024, to November 22, 2024). The data were collected under varying production and control conditions within the VRM's normal operating envelope. The aim was to investigate how operational variables influence the principles of material transfer within the mill. This dataset comprises 1050 samples collected under various conditions. The plant has nine cement production lines with a daily capacity of 13,800 tons. Materials (clinker and gypsum, d_80_: 32 mm) are fed into the vertical roller mill (VRM). The mill features four rollers (two Master and two Support), a 3000 kW main drive, a table diameter of 4.6 m, a roller diameter of 2.51 m, and a capacity of 120 tons per hour. It was manufactured by LOESCHE from Germany. Table 1 presents the VRM characteristics. The dataset used in this study originates from the same industrial monitoring campaign reported in our previous studies on VRM operation^16–19^. Those studies focused on different response variables associated with grinding performance (working pressure, feed rate, differential pressure, and main drive power), whereas the present study investigates mill fan speed and power to analyze the ventilation-driven material transportation subsystem.

**Table 1:** VRM information in the Tehran Cement Plant.

Characteristic	Value
Type of circuit	Closed circuit in the dry process
Nominal capacity	120 t/day
Master roller diameters	2.51 m
Support roller diameter	1.2 m
Table diameters	4.6 m
Main drive power	2880 kW
D_80_ of feed	32 mm

**Fig. 2:**
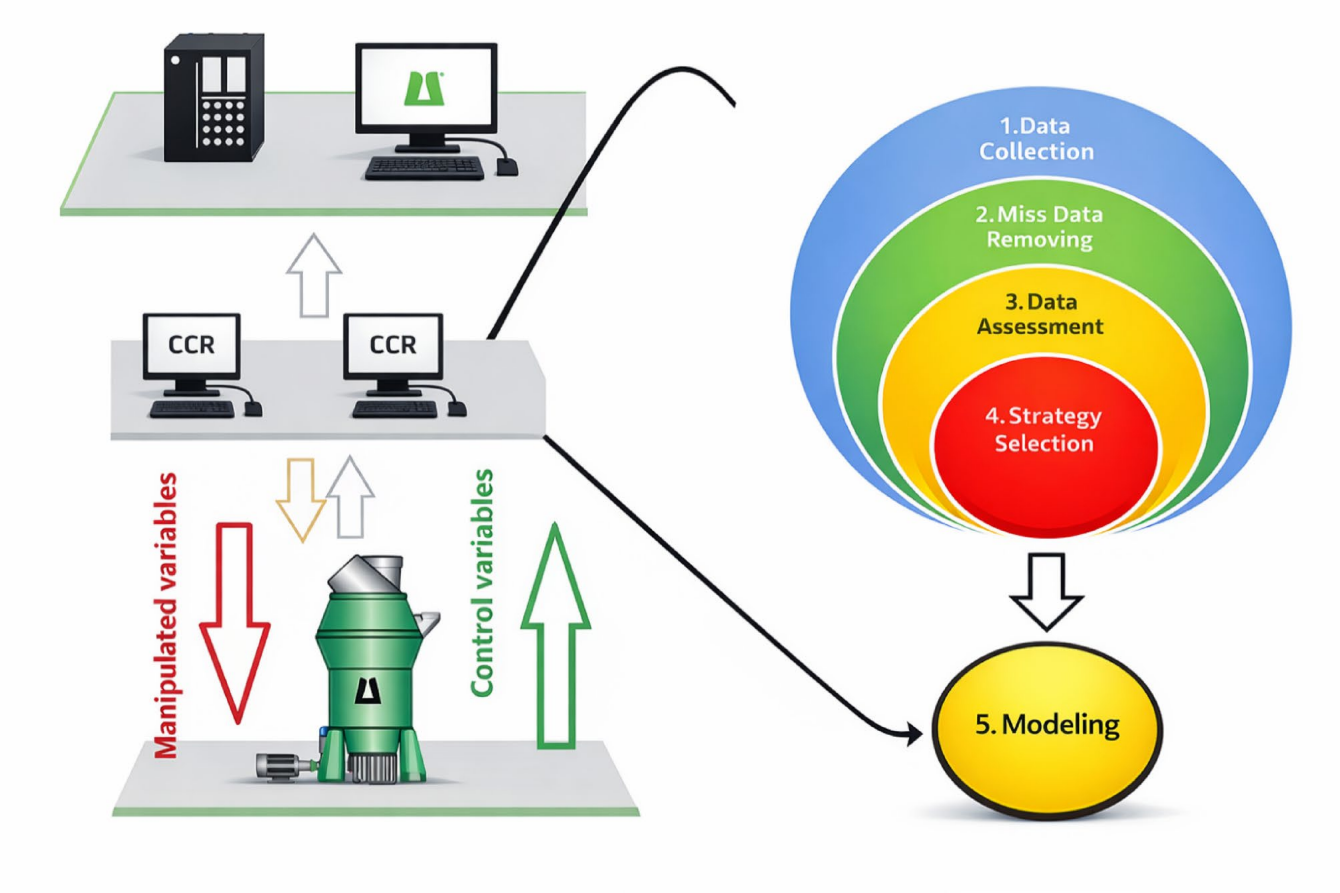
Schematic of the dataset collection from the VRM grinding circuit at Tehran Cement Plant.

The critical operational parameters of the VRM at Tehran Cement Plant were recorded to analyze material transport behavior inside the mill. The setpoints provided by the operators were treated as manipulated variables, while the actuator responses in the grinding circuit, recorded by the PLC, were treated as controlled variables. To establish and select the study strategy, the recorded process data were reviewed after each 12-h operational shift, and samples with missing values or outliers were identified and removed to ensure data quality before model development. Correlation analysis was then performed using a heatmap analysis. The relationship between mill fan operation and material transportation was modeled using several machine learning techniques, including XGBoost, CatBoost, and Random Forest. Figure 2 illustrates the schematic of the dataset collection from the VRM grinding circuit at Tehran Cement Plant. Temperature and humidity were not available as continuously monitored process variables in the plant database used for model development since they are approximately set in the plant.

The input variables used in this study were not selected through a priori feature screening. Instead, all operational variables that were continuously monitored and reliably recorded by the plant PLC system during the study period were included in the modeling dataset. Consequently, the subsequent SHAP analysis was used to identify and rank the relative importance of these variables rather than to validate a preselected subset.

### VRM

The working principle of VRMs relies on interparticle comminution, considered the most efficient breakage method according to Schönert^23^. During milling, the feed is initially directed to the center of the rotating table and then moved to the edges by centrifugal force. The material is compressed and ground between the rollers and the grinding table at the table's edges. After comminution, the broken particles are discharged over a dam ring, which controls the height of the material layer. These particles are then picked up by hot gas flowing from below the grinding table through a nozzle ring. The hot gas dries the material, and the finer particles are carried to a dynamic classifier at the top of the mill. The fine product is present in the gas and is collected in a filter^24,25^. Manipulating and controlling the grinding process in a VRM is accomplished by adjusting various online parameters, such as grinding pressure, feed rate, dp, etc. (Table 2).

**Table 2:** Descriptive of the operational variables of VRM.

Variables	Description of variables	Max.	Min.	Mean	STD
Inputs					
Feed rate (ton/h)	A load of mill feed	129.7	86.41	114.08	9.51
Working pressure (bar)	The applied pressure for grinding by the master roller	109.72	77.36	101.31	10.3
Counter pressure (bar)	The applied pressure for the adjustment of the distance of the master roller from the table	16.90	14.35	15.29	0.61
Water injection (m^3^/h)	The rate of water spray for stabilization of bed breakage	1.51	1.25	1.43	0.07
Differential pressure (mbar)	The differential pressure between the inlet and outlet of the mill	36.61	30.62	33.23	0.92
Mill body vibrating (mm/s)	The vibration of the mill body due to operational parameters	8.84	2.31	4.12	0.89
Main Drive (Kw)	The power drawn by the main motor	1887.80	1005.10	1552.713	224.24
Mill fan power (Kw)	The power drawn by the motor mill fan	677.44	513.38	555.77	19.79
Silo Elevator (A)	The amper drawing of the main elevator motor	64.71	44.49	55.38	4.70
Outputs					
Mill output pressure (mbar)	The outlet pressure of the mill	− 27.95	− 33.04	− 29.33	0.61
Mill fan speed (rpm)	The speed of the mill fan impeller	813.27	747.99	767.32	9.04

### Conscious Lab

The Conscious Lab (CL) concept is a data‑driven framework that uses pre‑collected industrial monitoring data to build explainable AI models for improving process understanding and supporting operational decision‑making^16,26–30^. In fact, the CL framework extends beyond conventional machine learning by establishing an explainable, data-driven virtual laboratory for industrial processes. Integrating predictive modeling with SHAP-based interpretability, it enables structured analysis of variable interactions and supports informed operational decision-making without additional physical experimentation. In this study, Extreme Gradient Boosting (XGBoost), CatBoost, Random Forest (RF), and Shapley additive explanations (SHAP) were used to build a *CL* model for predicting mill fan power and speed.

#### Extreme gradient boosting

Chen and Guestrin (2016) introduced Extreme Gradient Boosting (XGBoost), a robust and highly scalable tree-based boosting algorithm^31^. XGBoost is an ensemble method based on decision trees. It combines multiple weak regression trees sequentially, thereby improving their predictions and overall accuracy^32,33^. XGBoost differs from Gradient Boosted Decision Trees (GBDT) in several aspects. However, while GBDT uses only a first-order Taylor expansion, XGBoost incorporates a second-order Taylor expansion in its loss function. Furthermore, XGBoost normalizes the objective function to reduce model complexity and mitigate the risk of overfitting^34–36^. XGBoost enables parallel and distributed computation, leveraging the CPU's multithreading capabilities to accelerate the computation process^32,37^. XGBoost output can be computed as follows:

1 $${\widehat{y}}_{\mathrm{i}}=\sum_{k=1}^{k}{f}_{k}\left({X}_{i}\right),{f}_{k}\in\Gamma $$

where ${\widehat{{\boldsymbol{y}}}}_{\mathbf{i}}$ represents the predicted output, x refers to the input vector, ${{\boldsymbol{f}}}_{{\boldsymbol{k}}}$ indicates the output from the kth tree, and ${\boldsymbol{\Gamma}}$ signifies the function space encompassing all possible regression trees^38,39^. XGBoost's objective function is as follows:

2 $$\upphi =\sum_{i=1}^{n}l\left({y}_{i},{\widehat{y}}_{\mathrm{i}}\right)+\sum_{j=1}^{k}\Omega \left({f}_{j}\right)$$

where $l\left({y}_{i},{\widehat{y}}_{\mathrm{i}}\right)$ quantifies the discrepancy between the actual target ${y}_{i}$ and the predicted value ${\widehat{y}}_{\mathrm{i}}$ , $\Omega \left({f}_{j}\right)$ represents the regularization term that penalizes the model's complexity to prevent overfitting, and is determined by Eq. (3).

3 $$\Omega \left(f\right)=\gamma T+\frac{1}{2}\lambda \Vert \omega \Vert $$

where *T* represents the number of leaf nodes, and *ω * denotes the score associated with each leaf. The terms *γ * and *λ * are constant coefficients where *γ * refers to the complexity cost, and *λ * serves as a regularization hyperparameter used to control the extent of regularization^40,41^.

#### CatBoost

CatBoost is an advanced version of the gradient-boosting decision tree algorithm introduced by Prokhorenkova et al. in 2018^42^. Its main idea is to build a powerful regressor by iteratively combining weak regressors^43^. CatBoost exhibits powerful learning abilities for handling highly nonlinear datasets. Its training process involves optimizing the expected loss function (L(.)) using gradient descent as follows:

4 $${\mathrm{h}}^{\mathrm{t}}=\mathrm{a}\mathrm{r}\mathrm{g}\underset{\mathrm{h}\in \mathrm{H}}{\mathrm{min}}{\mathbb{E}}\left\{\mathrm{L}\left(\mathrm{y},{\mathrm{F}}^{\mathrm{t}-1}\left(\mathrm{X}\right)+\mathrm{h}\left(\mathrm{X}\right)\right)\right\}$$

where *y* represents the output, and *h* refers to a gradient step function chosen from H, ie, a family of functions. The step function is determined using the following:

5 $$\mathrm{h}(\mathrm{X})=\sum_{\mathrm{j}=1}^{\mathrm{J}}{\mathrm{b}}_{\mathrm{j}}{\mathbb{I}}_{\left\{\mathrm{X}\in {\mathrm{R}}_{\mathrm{j}}\right\}}$$

where ${R}_{j}$ represents the mutually exclusive regions corresponding to the leaves of the tree, ${b}_{j}$ is the predicted value for each region, and ${\mathbb{I}}$ serves as an indicator function. The expectation in Eq. 4 is estimated using the same dataset that introduces prediction shift and gradient bias. These problems can lead to overfitting. CatBoost addresses these challenges by using ordered boosting, thereby enhancing the model's robustness and generalization^37,44^.

#### Random forest

Breiman introduced Random Forest (RF), a nonparametric ensemble regression technique, in 2001^45^. RF is an extension of bagging that constructs a group of k randomized regression trees and takes their average. It integrates the random subspace approach with the bagging technique^46^. The features with the lowest relevance can be minimized using RF by evaluating their importance^47^. Using RF for regression problems offers several advantages: it has a low bias, requires only a small number of hyperparameters, and significantly reduces the risk of overfitting^48^. RF constructs an ensemble of K decision trees (DTs) as base models. Each data team (DT) makes its predictions independently, and the final output is obtained by averaging the individual predictions of all teams.

6 $$\widehat{T}(\mathrm{X})=\frac{1}{N}\sum_{k=1}^{K}{\widehat{T}}_{\mathrm{k}}\left(X\right)$$

where x denotes input and ${\widehat{T}}_{k}(X)$ is the estimation produced by the *k*th tree^41^.

#### Shapley additive exPlanations

Shapley additive explanations (SHAP), introduced by Lundberg and Lee in 2017, is an approach based on additive feature attribution^49^. The SHAP framework, rooted in game theory, offers a cohesive method for explaining the predictions of machine learning models while attributing importance to input features^44,50^. This approach represents the model's output as a linear combination of input variables. Specifically, the SHAP explanation model $g\left({Z}^{\prime}\right)$ for a prediction $f\left(X\right)$ is constructed by breaking down the prediction into a linear function of binary variables ${Z}^{\prime}\epsilon {\left\{\mathrm{0,1}\right\}}^{M}$ and SHAP values ${\phi}_{i}\epsilon {\mathbb{R}}$ , as shown in Eq. 7^51^.

7 $$f\left(X\right)=g\left({Z}^{\prime}\right)={\phi}_{0}+\sum_{i=1}^{M}{\phi}_{i}{Z}_{i}^{\prime}$$

where *M* represents the total number of input variables, ${Z}^{\prime}$ stands for the simplified input, and ${\phi}_{0}$ is a constant value when all features are absent. SHAP values indicate the direction and magnitude of each feature's impact on the model’s output^52,53^. SHAP values indicate the direction of each feature's effect on the model's output, and they can be derived as a potential solution to Eq. 7, adhering to three key properties: local accuracy, missingness, and consistency. These values can be computed as follows:

8 $${\phi}_{i}\left(f,X\right)=\sum_{{{Z}^{\prime}}^{\prime}\subseteq {X}^{\prime}}\frac{\left|{Z}^{\prime}\right|!\left(M-\left|{Z}^{\prime}\right|-1\right)!}{M!}\left[{f}_{X}\left({Z}^{\prime}\right)-{f}_{X}\left({{Z}^{\prime}}^{\vee }\right)\right]$$

where ${X}^{\prime}$ represents the vector containing the selected *M* input variables, *f* refers to the trained model,$\left[{f}_{X}\left({Z}^{\prime}\right)-{f}_{X}\left({{Z}^{\prime}}^{\vee }\right)\right]$ captures the contribution of the *i* th variable to the model’s output and $\left|{Z}^{\prime}\right|$ indicates the count of non-zero elements in ${Z}^{\prime}$^51,54^.

#### Model evaluation

The model's accuracy was evaluated using several metrics: the coefficient of determination (R^2^), root mean square error (RMSE), and differences between actual and predicted values at various stages of the modeling process (including training, testing, and validation). The Pearson correlation coefficient (R^2^) measures the linear relationship between variables, with values ranging from -1 to + 1. Its sign indicates the direction, and its magnitude reflects the strength of the correlation. The coefficient of determination (R^2^), also known as the “goodness of fit,” measures model accuracy; values closer to 1 indicate greater reliability. R^2^ represents the proportion of variance in the output explained by the input variables. Finally, RMSE quantifies the difference between actual and predicted values, assessing the model’s prediction error^55–57^. Based on the expected values ${\widehat{y}}_{n}$ of a regression’s dependent variable ${y}_{n}$ across time points (n), RMSE as follows:

9 $$RMSE=\sqrt{\frac{\sum_{i=n}^{N}{({\widehat{y}}_{n}-{y}_{n})}^{2}}{N}}$$

Furthermore, to achieve more reliable model validation, the nested cross-validation (NCV) method was employed. This approach was first introduced by Stone in 1974^58^. Nested cross-validation (NCV) was used to reduce bias in estimating model performance. In this approach, the dataset is divided into training and validation sets in an outer loop, while an inner cross-validation loop is applied to the training data for hyperparameter tuning. The final model performance is then evaluated on the independent outer validation set, providing a reliable estimate of performance on unseen data^58,59^. Although the dataset was collected over an eight-month period, the present study was not designed as a time-series forecasting problem. Instead, each observation represents a distinct operating state characterized by a unique combination of process measurements and operator-defined control settings. The objective of the proposed framework is to learn relationships among operational variables within the VRM operating domain, rather than predict future observations from historical sequences. Consequently, the developed models are process-state prediction models rather than temporal forecasting models, and random partitioning within the NCV framework was therefore adopted.

## Results and discussion

### Linear correlations

**Fig. 3:**
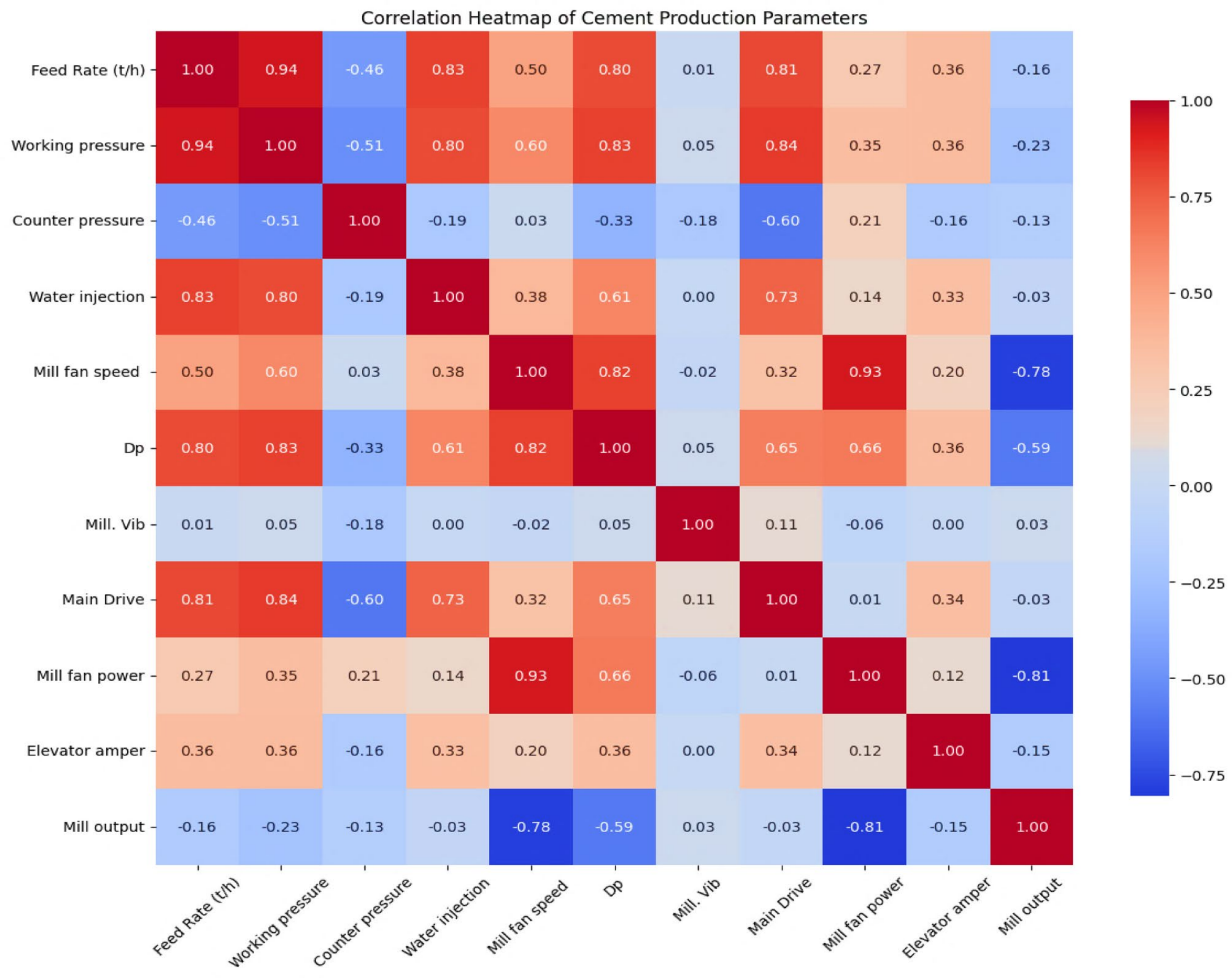
Pearson correlation between VRM operational parameters.

Evaluating the relationships among variables to rank them by effectiveness can offer valuable insights into the model and clarify how it functions. Pearson correlation and SHAP analyses were used to assess relationships across the entire dataset. To investigate the relationship between the operational parameters of the clinker grinding circuit in the cement VRM, the Pearson correlation coefficient was employed. A heat map illustrates the relationships between various mill variables. VRM operates in a dry system, where the mill fan transports powdered materials^4,60^. Based on experience with cement VRMs, in addition to fan speed, other parameters also influence material transportation in these mills^2^. Figure 3 illustrates the positive and negative correlations and impacts between the variables. The mill fan speed exhibits the highest correlation with mill fan power and dp, with correlation coefficients of 0.93 and 0.82, respectively. Conversely, it shows the strongest negative correlation with mill output pressure, at -0.78. It is evident that when controlling the cement roller mill, as the operator increases the mill fan speed, the mill differential pressure also increases, resulting in higher mill fan power consumption (positive correlation), while the mill output pressure becomes increasingly negative (negative correlation).

### Variable ranking assessments by SHAP

**Fig. 4:**
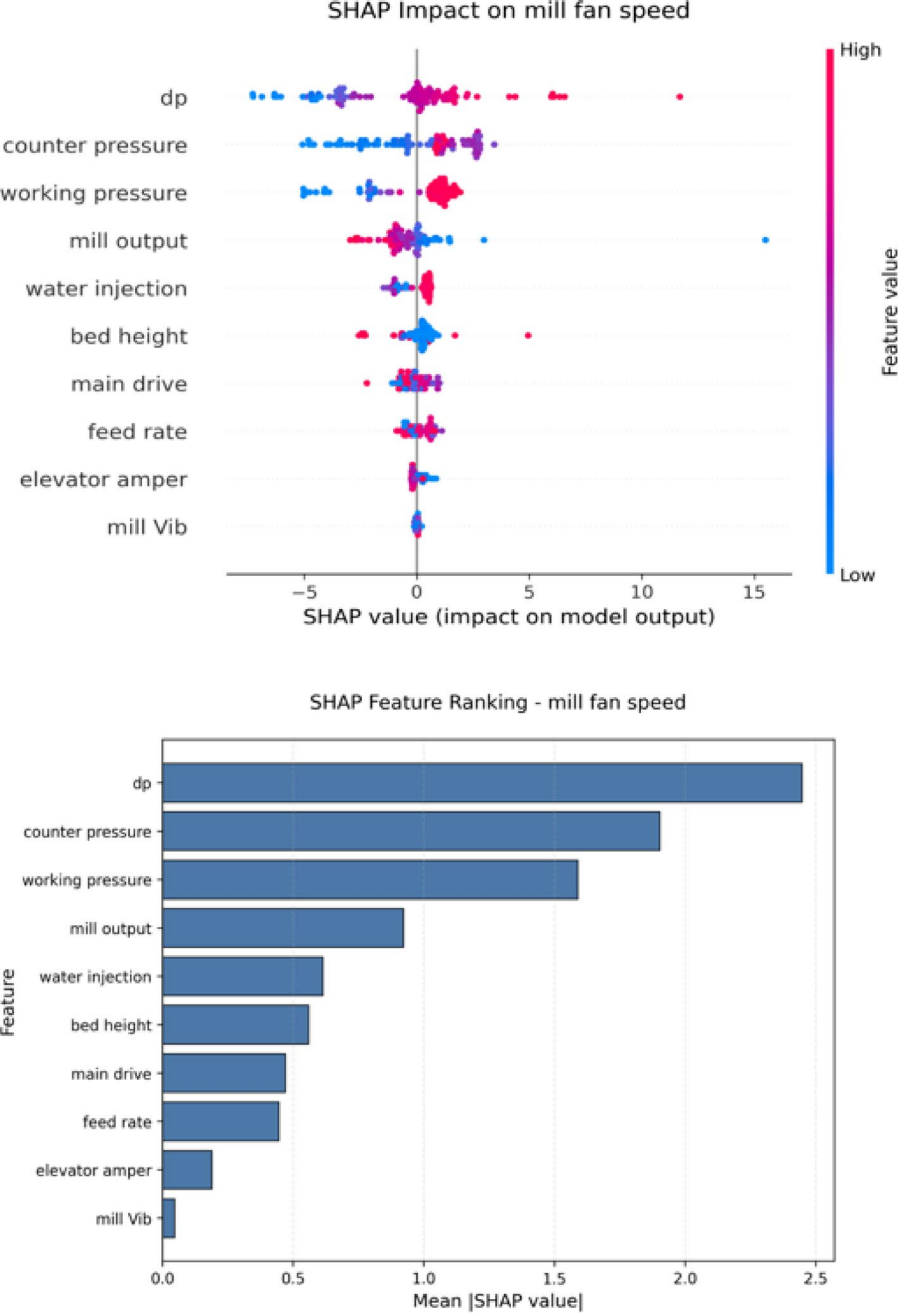
Ranking variables based on their mean SHAP-CatBoost value and relationship magnitude for Mill fan speed prediction.

**Fig. 5:**
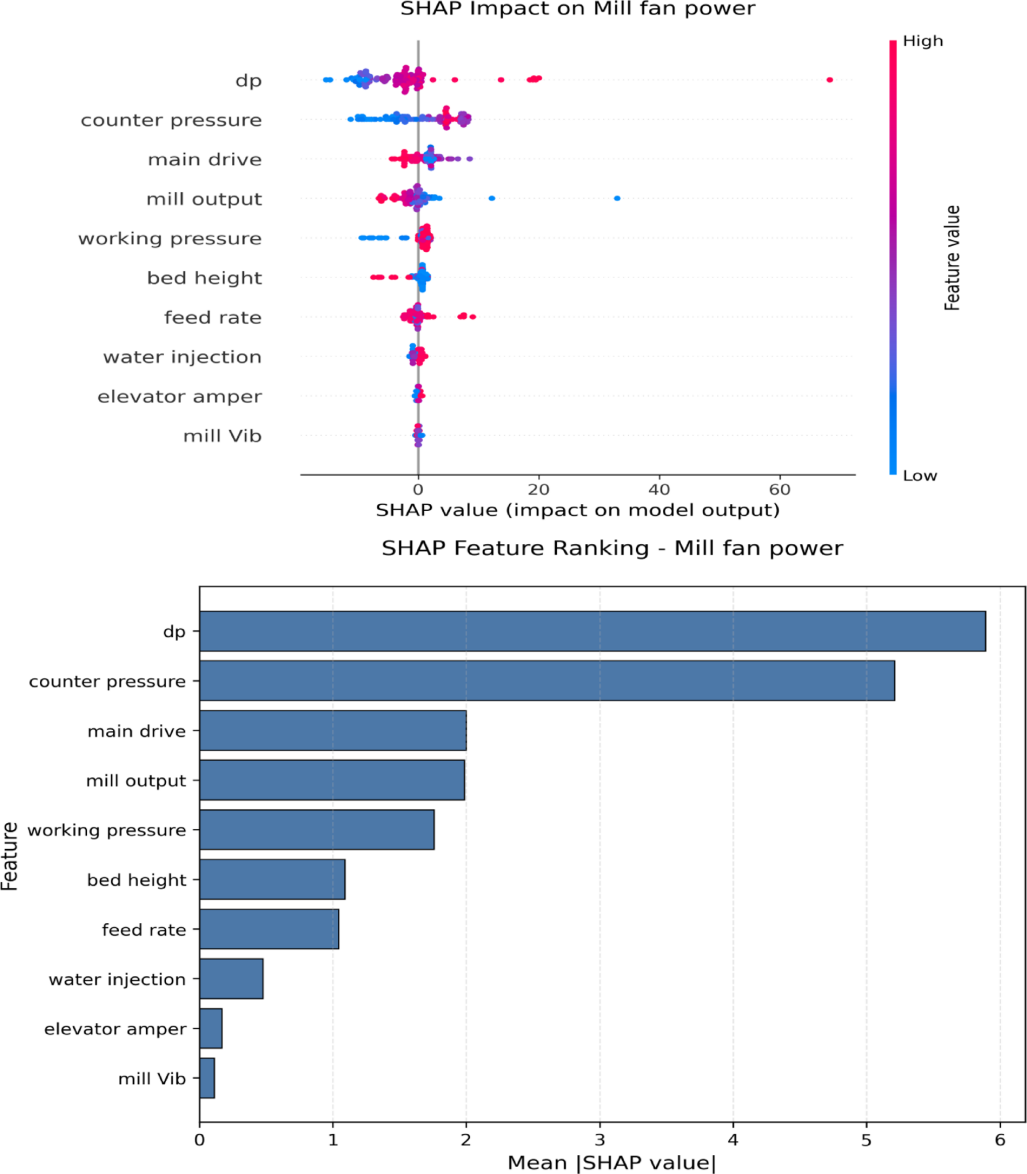
Ranking variables based on their mean SHAP-CatBoost value and relationship magnitude for Mill fan power prediction.

SHAP analyses in Figs. 4 and 5, corresponding to mill fan speed and fan drive power modeling, assess the relationships among operational variables across the entire dataset. The results showed that, in order of importance, the most important variables predicting mill fan speed for the transfer of ground material are dp, counter pressure, working pressure, and mill output pressure. On the other hand, for predicting fan drive power, which is entirely related to the mill fan speed, the most important variables, in order, are dp, counter pressure, main drive, and mill output pressure. The meaningful relationship between the case where the fan speed is the target and the case where the mill fan power is the target is as follows: fan speed is directly related to its power drawing, meaning that any change in fan speed will lead to a change in power^61^, and as a result, the operator will need to adjust the required working pressure (Fig. 4). The SHAP results (Figs. 4 and 5) indicate that three influential parameters namely dp, counter pressure, and mill output pressure are common to both modeling targets, mill fan speed and mill fan power. This suggests that these variables play a fundamental role in controlling both material transportation performance and fan power demand. In practice, the operator adjusts the fan speed, thereby changing the fan power consumption, to regulate the dp and the mill outlet pressure. It is evident that variations in fan speed typically occur simultaneously with changes in counter pressure to adjust the distance between the rollers and the material bed. In addition, adjusting the working pressure affects the mill's main drive power. Therefore, the presence of these variables in the SHAP ranking for both target fan speed and fan power reflects the operational and control relationships among these parameters in the mill operation process^62^.

**Fig. 6:**
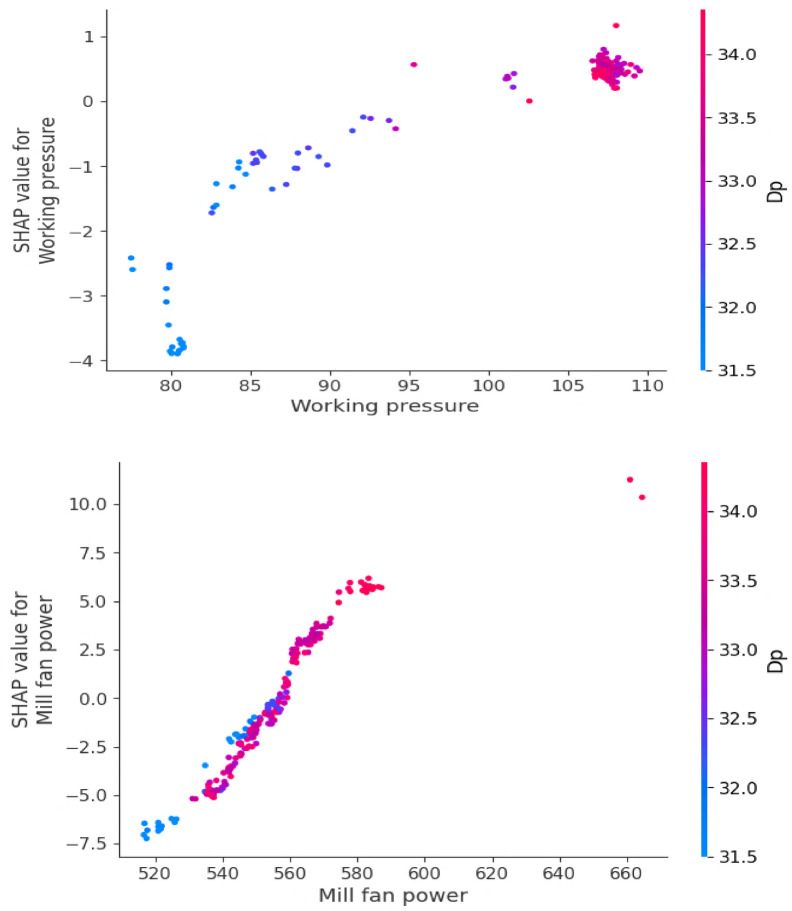
SHAP values for multi-interactions between VRM grinding circuit operational variables (Part A).

In the mill's operational state, the SHAP approach identifies multiple nonlinear correlations among operational parameters that affect the target. The heatmap in Fig. 3 demonstrates a positive linear correlation between the mill fan speed (as the model target) and the variables, including mill fan power, mill dp, working pressure, and main drive power, with values of 0.93, 0.82, 0.60, and 0.32, respectively (Fig. 3). In addition. At the same time, the SHAP approach captures multiple correlations. Figure 6 examines SHAP values and multicollinearity among mill dp, working pressure, and mill fan power. The SHAP values exhibit a negative interaction correlation at working pressures below approximately 95 bar, transitioning to a positive correlation at pressures above 95 bar. The trend of intercorrelation shifts from negative to positive as the mill working pressure increases, because the operator increases mill feed rates when the working pressure exceeds 95 bar. Naturally, an increased feed rate leads to a rise in the differential pressure. Further analysis of the interaction correlation between d_P_ and mill fan power indicates a shift from negative to positive at a mill fan power of 550 kW. This shows that d_P_ will also increase for mill fan power values above 550 kW, with the reverse trend holding.

**Fig. 7:**
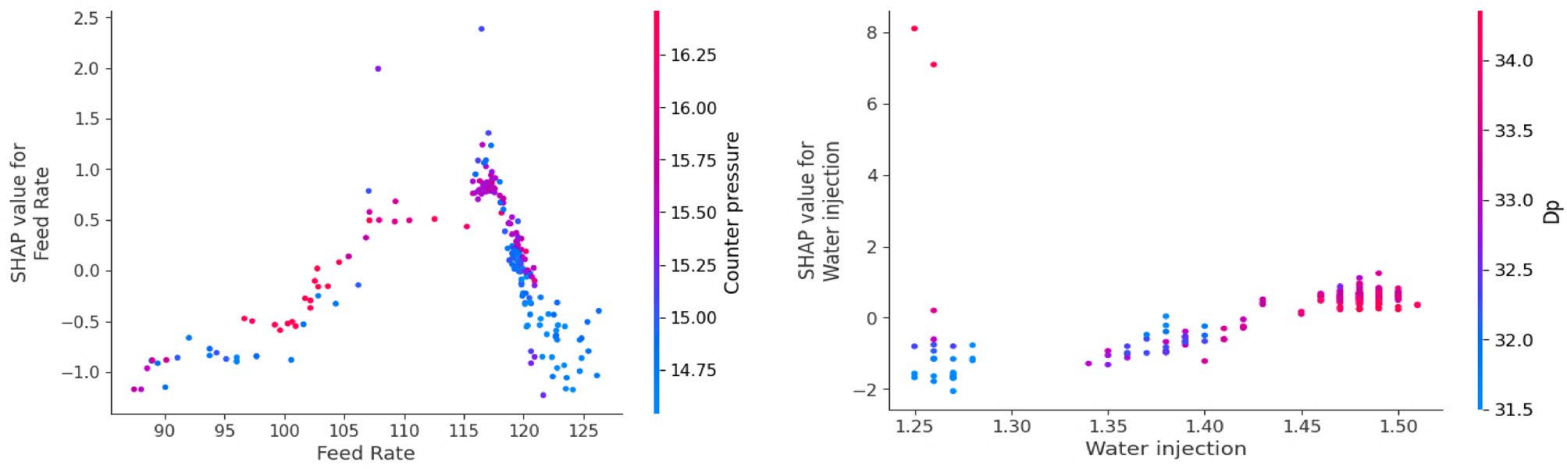
SHAP values for multi-interactions between VRM grinding circuit operational variables (Part B).

The assessment of the interaction correlation between feed rate and counter pressure in mill fan speed modeling is shown in Fig. 7. The Pearson correlation between these parameters (Fig. 3) is − 0.46. However, SHAP analysis indicates a positive correlation within the 103–118 t/h range, while correlations are negative below and above this range. The negative correlations are due to an increase in feed rate and a simultaneous decrease in counter pressure. In this state, the operator reduces the counter pressure (bringing the rollers closer to the material bed surface for better grinding), which consequently leads to an increase in working pressure as the counter pressure drops (based on Fig. 3, the correlation with working pressure is − 0.51, and it is − 0.46 with feed rate). Within the positive correlation range, the feed rate is accompanied by an increase in counter pressure, resulting in an appropriate material bed thickness for grinding. The linear correlation between d_P_ and water injection is 0.61. However, SHAP analysis shows interaction correlation values above 1.45 for water injection. The positive correlation in this range is due to increased water injection, which creates a more stable grinding bed, increases the average residence time of materials, and raises the dp. The negative correlation observed for values below 1.45 is due to the operator’s response to reduced dp (Fig. 7). The operator increases water injection to counteract the decreasing dp trend.

### Material transportation prediction

#### Mill fan speed and Mill fan power predictions

The data used in this study, consisting of operator-set setpoints via the Programmable Logic Controller (PLC) and actuator responses in the grinding circuit, were randomly divided into three subsets: 80% for training, 10% for testing, and 10% for validation. This partitioning was performed to develop and evaluate models for predicting mill fan speed and power. During validation, the optimal hyperparameters for CatBoost, XGBoost, and Random Forest (RF) were determined. The validation set was used to explore various hyperparameter values via a trial-and-error (tuning) process to identify the best set (Table 3).

**Table 3:** The parameter settings for the predictive models.

	Algorithms		
XGBoost	CatBoost	XGBoost	RF
Learning rate	0.1	0.1	–
Maximum number of trees	1000	1000	1000
Maximum depth of trees	6	6	–
*γ *	–	1	–

Table 4 shows the MSE, RMSE, and R^2^ for the modeling stages using all three mentioned algorithms and linear regression. Linear regression models were developed using the same data partitions employed for the machine-learning models. In contrast, CatBoost addresses the prediction shift using an ordered boosting approach. It also mitigates gradient-boosting biases by utilizing oblivious decision trees and implementing a consistent splitting criterion at each tree level. Research by Bentjejac et al., Gao et al., and Ibrahim et al. has demonstrated that the CatBoost algorithm outperforms other machine learning algorithms, such as Random Forest (RF) and Support Vector Regression (SVR), in regression tasks^63–65^. The results of SHAP-CatBoost modeling demonstrate the principle of material transportation, which relates to fan speed and energy consumption and is linked to the fan power required by a VRM. This modeling approach is a powerful AI-based system grounded in practical experience with these mills and the knowledge of the grinding process.

10 $$ \begin{aligned} {\text{Mill fan power}} = & - {224}.{822148} - 0.{668956}\left( {\text{feed rate}} \right) + {1}.{7}0{4148}\left( {\text{working pressure}} \right) \\ & + {1}0.{9419}0{2}\left( {\text{counter pressure}} \right) - 0.0{54654}\left( {\text{bed height}} \right) \\ & - {78}.{11}0{789}\left( {\text{water injection}} \right) + {13}.{316}0{24}\left( {{\mathrm{dp}}} \right) - 0.0{35367}\left( {\text{mill Vib}} \right) \\ & - 0.0{4}0{938}\left( {\text{main drive}} \right) - 0.{168634}\left( {\text{elevator amper}} \right) - {8}.{853321}\left( {\text{mill output}} \right) \\ \end{aligned} $$

11 $$ \begin{aligned} {\text{Mill fan speed}} = & \, {4}0{4}.{958343} - 0.{454626}\left( {\text{feed rate}} \right) + 0.{815948}\left( {\text{working pressure}} \right) \\ & + {4}.{545936}\left( {\text{counter pressure}} \right) - 0.0{35218}\left( {\text{bed height}} \right) \\ & - {23}.{267114}\left( {\text{water injection}} \right) + {6}.{1215}0{5}\left( {{\mathrm{dp}}} \right) - 0.0{19958}\left( {\text{mill Vib}} \right) \\ & - 0.00{6516}\left( {\text{main drive}} \right) - 0.{118681}\left( {\text{elevator amper}} \right) - {3}.{713326}\left( {\text{mill output}} \right) \\ \end{aligned} $$

**Table 4:** Outcomes of modeling stages for predicting mill fan speed and power.

Mill fan power	Train			Validation			Test		
Method	R^2^	RMSE	MSE	R^2^	RMSE	MSE	R^2^	RMSE	MSE
Linear regression (Eq. 10)	0.87	7.08	50.19	0.83	7.77	60.51	0.85	6.85	47.05
CatBoost	0.9835	2.5729	6.6201	0.9548	4.0703	16.5675	0.9480	4.1834	17.5011
XGBoost	0.9802	2.8220	7.9639	0.9356	4.8591	23.6110	0.9425	4.4804	20.0745
Random Forest	0.9835	2.5729	6.6201	0.9248	5.0564	25.5675	0.9401	4.8487	23.5011

Mill fan speed	Train			Validation			Test		
Method	R^2^	RMSE	MSE	R^2^	RMSE	MSE	R^2^	RMSE	MSE
Linear regression (Eq. 11)	0.91	2.67	7.16	0.87	3.07	9.43	0.90	2.70	7.32
CatBoost	0.9971	0.4928	0.2429	0.9743	1.4887	2.2162	0.9781	1.2789	1.6355
XGBoost	0.9945	0.6738	0.4540	0.9712	1.4631	2.1407	0.9703	1.3840	1.9155
Random Forest	0.9905	0.8018	0.6429	0.9693	1.4887	2.2162	0.9601	1.4613	2.1355

Modeling outcomes indicated that CatBoost achieves the highest R^2^ for both Mill fan power and speed. The prediction accuracies for mill fan speed and mill fan power were 0.97 and 0.95, respectively. The MSE, RMSE, and R^2^ values for different stages are presented, indicating the superiority of CatBoost, although the predictions from XGBoost and RF are also acceptable. Although the regression models captured the dominant linear relationships among the operational variables, their predictive performance was consistently lower than that of the ensemble learning algorithms. For example, linear regression yielded a test R^2^ of 0.85 for mill fan power prediction and 0.90 for mill fan speed prediction, whereas CatBoost achieved test R^2^ values of 0.948 and 0.978, respectively. These results indicate that explainable ensemble learning models better capture the nonlinear interactions among operational variables governing the material transportation process.

**Fig. 8:**
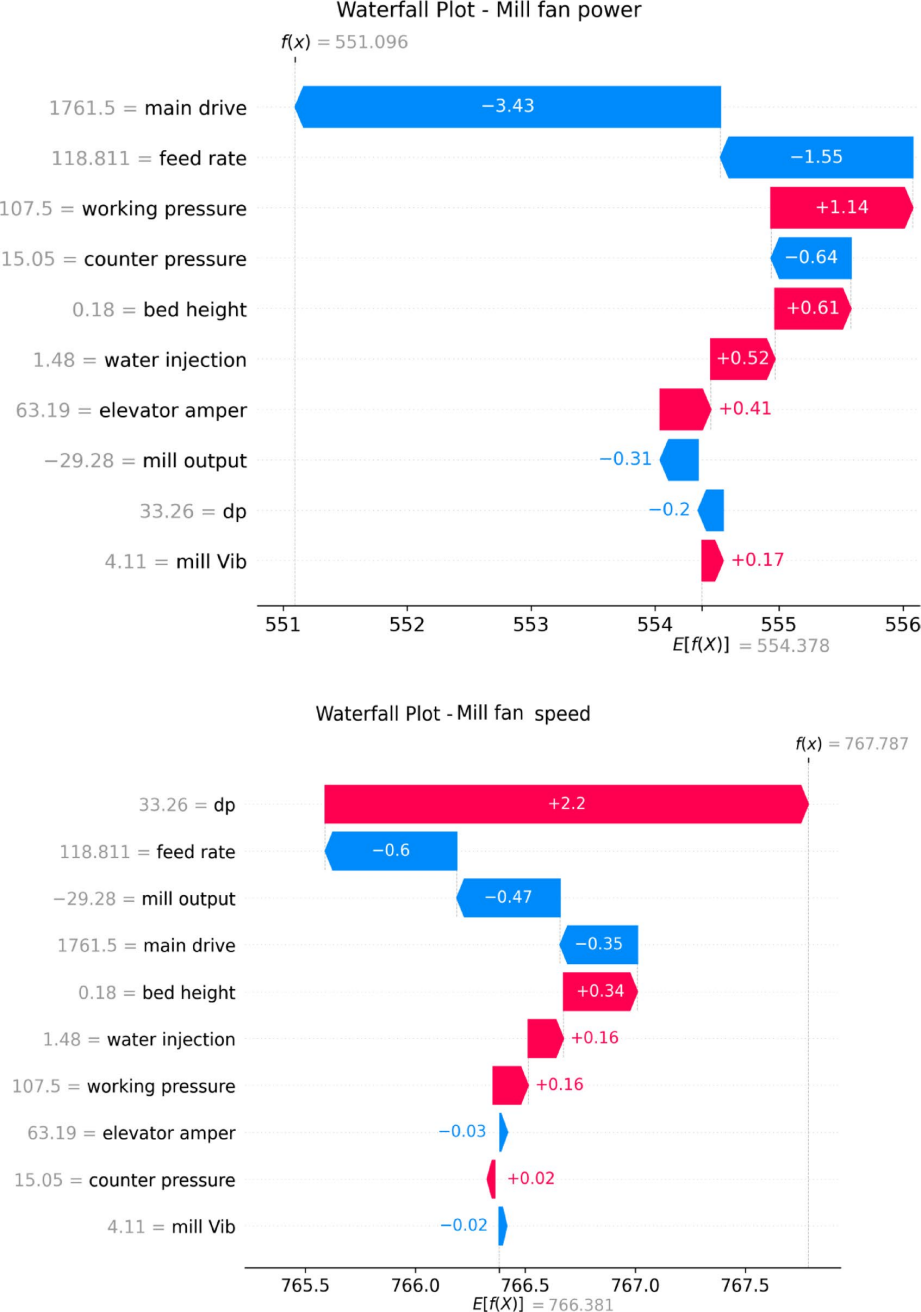
First instance of SHAP waterfall plot visualizing the predicted feature contributions to mill fan speed and power.

Figure 8 presents SHAP waterfall plots illustrating the predictions of mill fan power and speed for a specific instance using a CatBoost model. These plots quantify the contribution of each input feature to the final model output; red arrows denote features that exert a positive impact (increasing the prediction), while blue arrows signify features that have a negative impact (decreasing the prediction). In the mill fan power plot (Fig. 8), the base value is [Ef(x)] = 551.096, and the model yields a final prediction of 554.378 by aggregating the SHAP contributions from various operational parameters, such as the main drive and feed rate. Similarly, the mill fan speed plot illustrates the prediction process, starting with a base value of [Ef(x)] = 766.381 and arriving at 767.787. The vertical dashed line at the base value serves as a reference point, allowing for a clear visualization of how each variable deviates the prediction from the expected value. This methodological breakdown provides an interpretable assessment of the driving factors behind the mill’s operational performance, effectively highlighting the significance of variables such as main drive, dp, and feed rate in shaping the system’s dynamics.

Although the data were collected over several months, each row of the dataset represents a distinct operational condition of the VRM with different process settings rather than sequential time‑dependent measurements. Therefore, the samples were treated as independent observations, and random partitioning within the Nested Cross‑Validation (NCV) framework was considered appropriate for model evaluation. To enhance the accuracy and reliability of model evaluation, a 5 × 3-layer NCV approach was employed. This method, consisting of inner and outer loops, ensures a complete separation between training and validation data throughout all stages of parameter tuning and model development, thereby preventing evaluation bias. According to the results presented in Table 5, CatBoost demonstrated superior performance compared with XGBoost and Random Forest in predicting the two key targets: mill fan power and mill fan speed. For mill fan power, CatBoost achieved an R^2^ of 0.94 ± 0.01and an RMSE of 4.67 ± 0.43, and was therefore identified as the best-performing model. Although the performance difference from Random Forest was minimal, CatBoost was selected for its higher accuracy and stability. For mill fan speed prediction, CatBoost again produced the best results, achieving an R^2^ of 0.98 ± 0.005 along with the lowest RMSE of 1.34 ± 0.26. While XGBoost delivered reasonable performance, it consistently underperformed compared to the other two ensemble methods. Overall, these findings indicate the superior performance and strong generalizability of the CatBoost model in predicting both key variables. This was further validated using the Nested Cross‑Validation framework, highlighting CatBoost's significant potential to enhance the intelligent and stable operation of VRM systems.

**Table 5:** Nested Cross-Validation results (5 × 3-fold): CatBoost, XGBoost, RF on VRM targets.

Method	R^2^ (mean ± std)	RMSE (mean ± std)	MSE (mean ± std)
Mill fan power			
CatBoost	0.94 ± 0.01	4.67 ± 0.43	21.99 ± 4.22
XGBoost	0.92 ± 0.02	5.36 ± 0.85	29.53 ± 9.41
RandomForest	0.93 ± 0.01	4.73 ± 0.35	22.58 ± 3.41
Mill fan speed			
CatBoost	0.98 ± 0.005	1.34 ± 0.26	1.887 ± 0.71
XGBoost	0.96 ± 0.026	1.619 ± 0.57	2.955 ± 2.19
RandomForest	0.96 ± 0.005	1.376 ± 0.19	1.931 ± 0.51

**Fig. 9:**
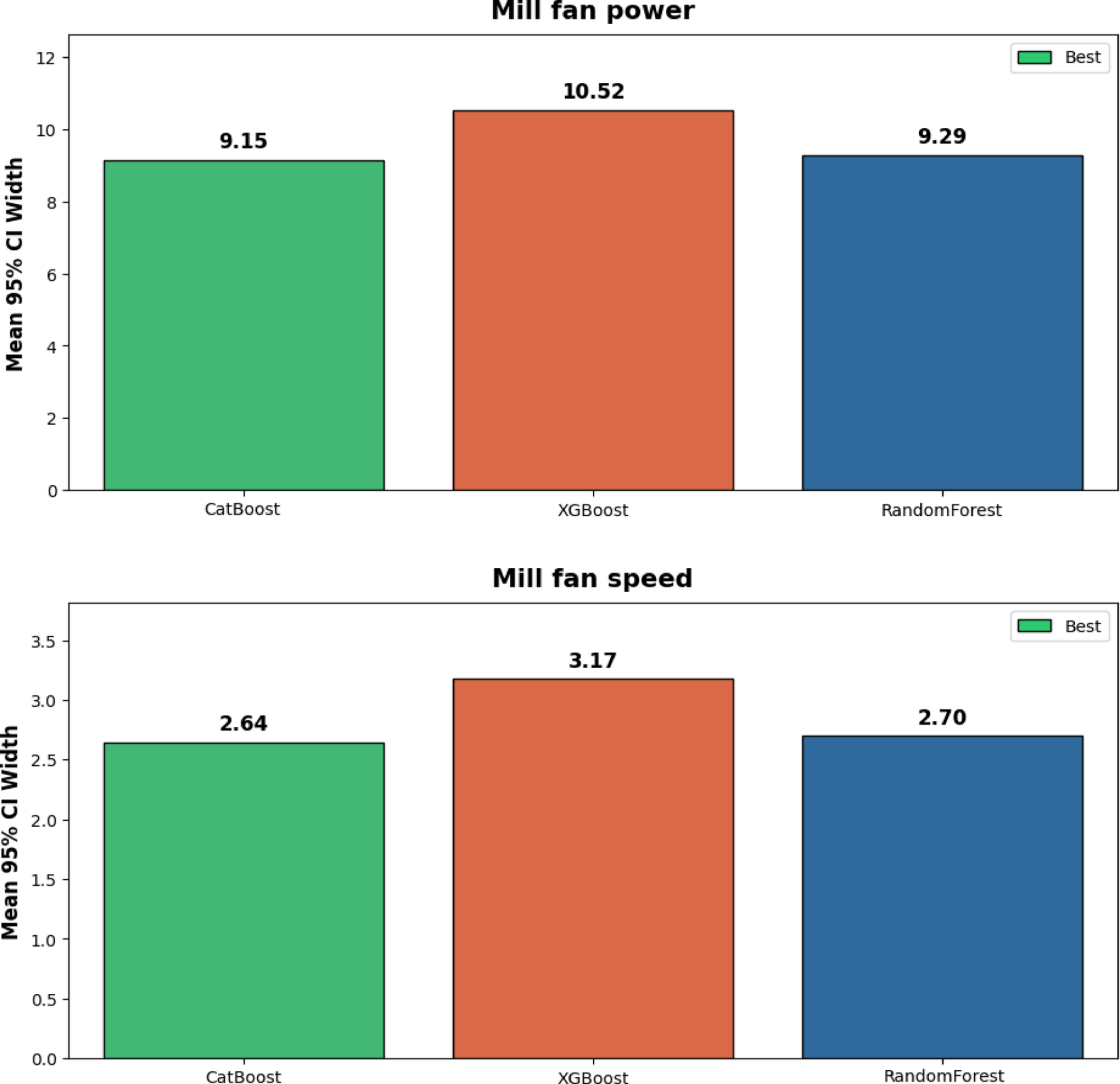
Mean 95% CI width for each model on fan power and speed via nested cross-validation.

As shown in Fig. 9 the mean width of the 95% confidence intervals for the CatBoost, XGBoost, and Random Forest models in predicting (top) mill fan power and (bottom) mill fan speed using nested cross-validation is reported. A narrower confidence interval indicates greater model stability and lower prediction uncertainty. CatBoost achieved the smallest CI width for both key variables, demonstrating greater reliability than the other evaluated models.

### Fundamental analysis of the transportation principles

**Fig. 10:**
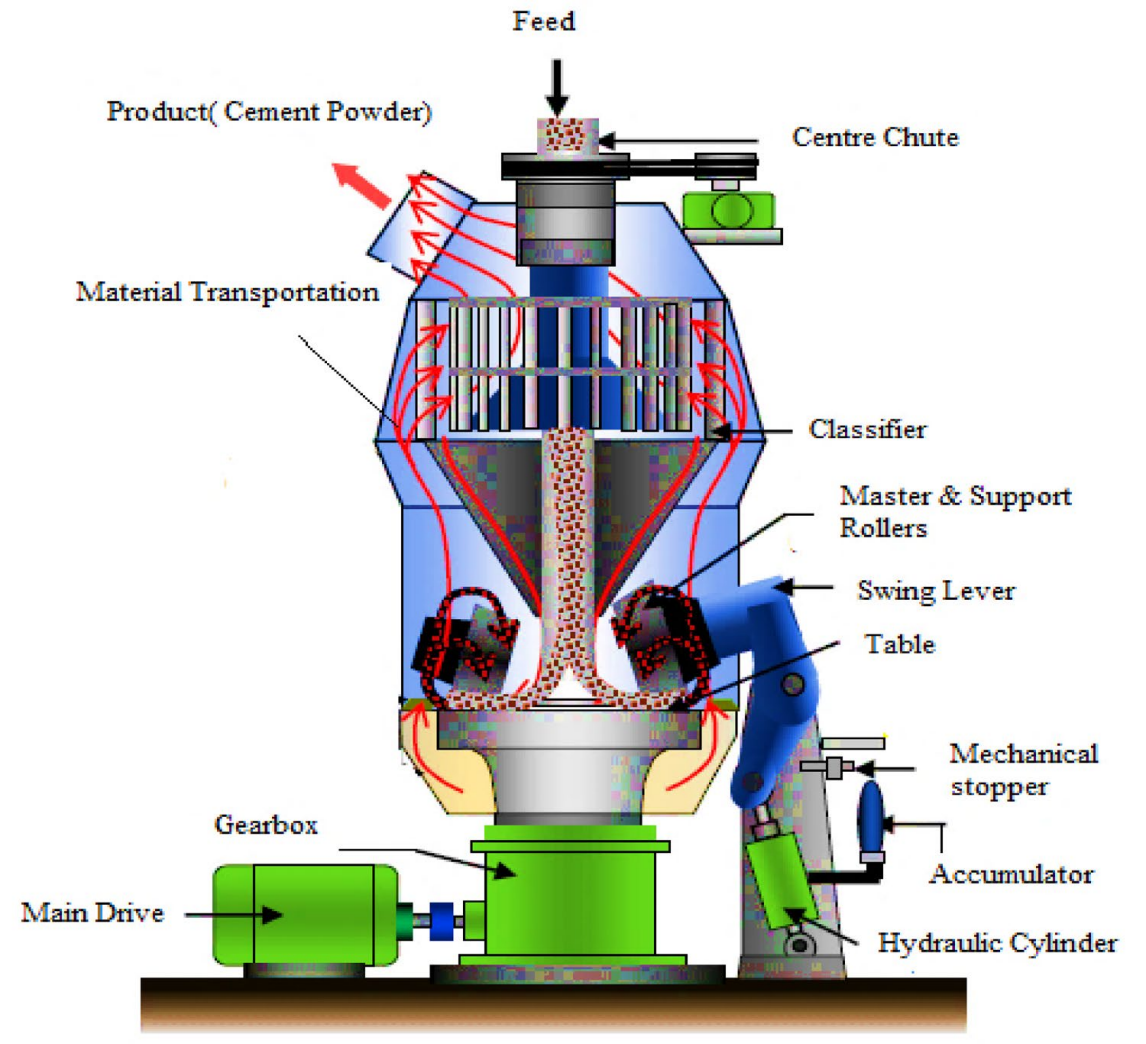
Schematic of a VRM based on the material-transportation principle.

The VRM simultaneously performs drying, grinding, classification, and pneumatic transportation^66^. Materials enter through the inlet at the center of the table and, as illustrated in Fig. 10, are transferred to the table's edge, where they are trapped between the table and the roller. The system allows precise control by adjusting the operating pressure. The ground materials are dried using hot gas, which the mill fan draws through the nozzle ring around the table's perimeter. The dried materials are then reported to the classifier, where they are separated into final products with the desired particle size^67–69^. The fan power increases with mill fan speed, resulting in a more negative outlet pressure for the mill^2,61^. This explains the high negative correlation between speed, power, d_P_, and mill outlet pressure^61^. Significant negative correlations enhance understanding of the transport of ground materials via the mill fan. Material transportation in roller mills is one of the four key principles of operation. Due to the short retention time in VRM, the number of breakage events is 2–3^12^. Therefore, if the transportation process is flawed, other effective process parameters are disrupted, reducing the mill's efficiency and causing multiple processing issues, such as mill body vibration^62^. Thus, developing artificial intelligence as a CL-based approach leverages expert knowledge and provides operators with a deep understanding of the importance of material transportation principles and the variables that influence this topic. As a result, grinding circuit efficiency and energy consumption improve, and the interaction with the grinding circuit becomes more intelligent.

**Fig. 11:**
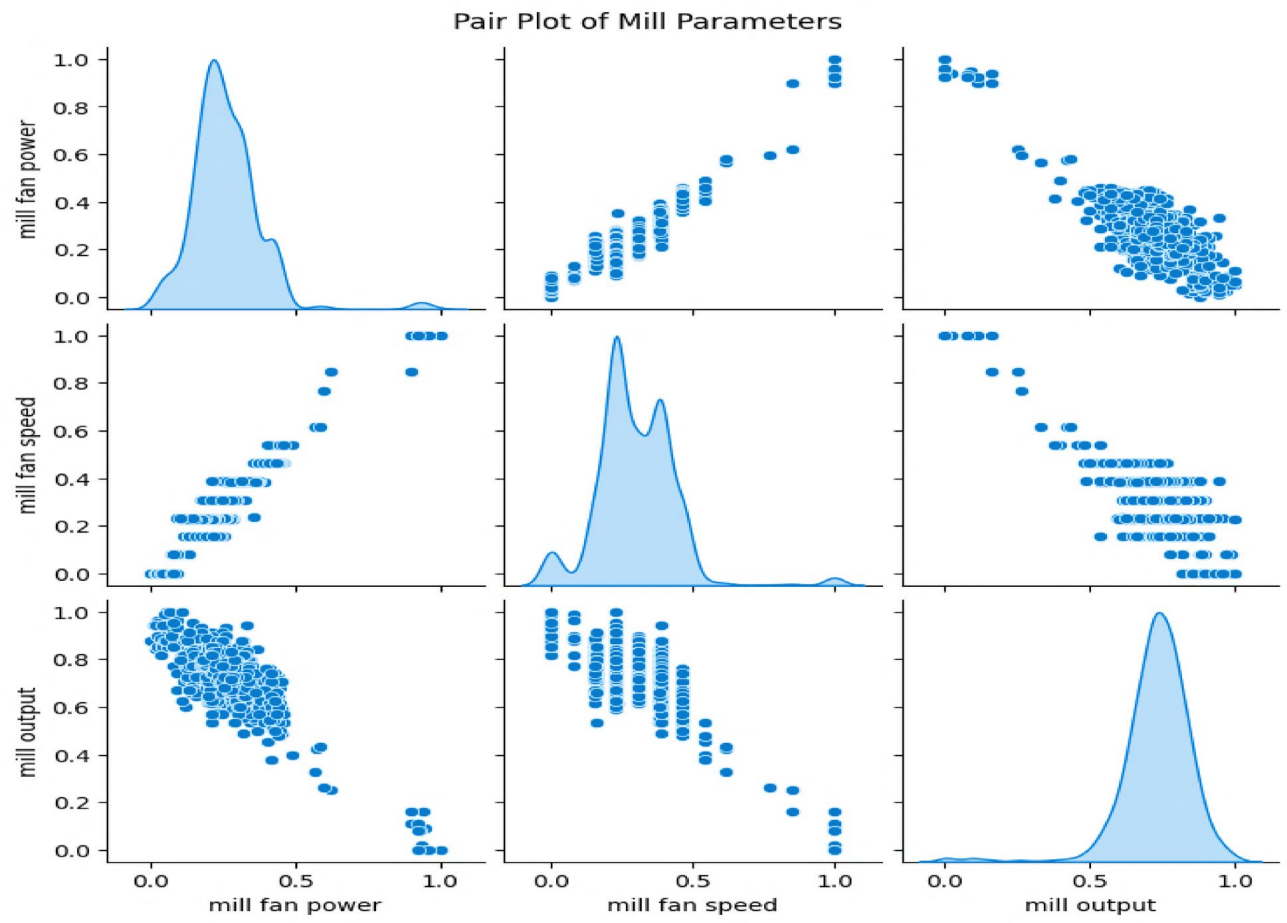
The Relationship Between Effective Parameters in Material Transport in VRM Circuits.

The relationship between the parameters affecting material transportation in a VRM for cement is clearly illustrated in Fig. 11. However, when the operator increases the fan speed, the fan power also increases. With increased fan power, the mill's outlet pressure becomes more negative, reducing the differential pressure. Therefore, the operator must choose the best alternative based on the feed conditions and other dependent parameters. Thus, the *CL* approach has opened a pathway towards intelligent operation, which can overcome operational issues.

## Conclusion

This work extends the previously established Conscious Lab framework to analyze ventilation-driven material transport in an industrial vertical roller mill. Using 1050 real industrial observations from the Tehran Cement Plant, models were developed to predict two ventilation-centered targets: mill fan speed and mill fan power, as these variables directly condition pneumatic conveyance, mill stability, and energy use in a dry VRM.

Across the evaluated algorithms, CatBoost provided the most consistent predictive capability for both targets (approximately ${R}^{2}\sim 0.97$ for fan speed and ${R}^{2}\sim 0.95$ for fan power on testing data), and nested cross-validation further supported its robustness. Correlation analysis and SHAP explanations jointly confirmed that ventilation-related variables dominate the transport response: fan speed/power and mill differential pressure were repeatedly identified as the most influential factors, while mill outlet pressure showed a strong inverse association with fan operation, consistent with the physical mechanism of induced draft and pneumatic lifting in VRMs. Beyond ranking, SHAP interaction patterns revealed that several relationships are regime-dependent (nonlinear), indicating that operator actions (e.g., coordinated adjustments of pressure, feed, and water injection) can shift transport behavior and stability across operating ranges.

In practice, the CL framework provides a transparent decision-support layer that helps operators understand *why* fan power/speed changes occur under varying mill conditions and which variables most strongly drive transport-related outcomes. As a next step toward deployment, future work should evaluate the approach under additional campaigns and incorporate explicit process constraints and control objectives (e.g., stability limits, vibration thresholds, product quality targets), enabling closed-loop or advisory control while retaining explainability. Overall, the study demonstrates that explainable machine learning trained on routine plant-monitoring data can meaningfully characterize VRM material transportation and support more stable, energy-aware operation.

## Data Availability

The datasets used during the current study are available from the corresponding author on reasonable request.
